# The complete N-terminal extension of heparin cofactor II is required for maximal effectiveness as a thrombin exosite 1 ligand

**DOI:** 10.1186/1471-2091-14-6

**Published:** 2013-03-07

**Authors:** Amanda J Boyle, Leigh Ann Roddick, Varsha Bhakta, Melissa D Lambourne, Murray S Junop, Patricia C Liaw, Jeffrey I Weitz, William P Sheffield

**Affiliations:** 1 Departments of Pathology and Molecular Medicine, McMaster University, 1280 Main Street West, Hamilton, ON L8S 4K1, Canada; 2 Department of Biochemistry and Biomedical Sciences, McMaster University, 1280 Main Street West, Hamilton, ON L8S 4K1, Canada; 3 Department of Medicine, McMaster University, 1280 Main Street West, Hamilton, ON L8S 4K1, Canada; 4 Department of Research and Development, Canadian Blood Services, HSC 4N66 McMaster University, 1280 Main Street West, Hamilton, ON L8S 4K1, Canada

**Keywords:** Heparin cofactor II, Thrombin, Serpins, Exosites, Coagulation, Inhibition

## Abstract

**Background:**

Heparin cofactor II (HCII) is a circulating protease inhibitor, one which contains an N-terminal acidic extension (HCII 1-75) unique within the serpin superfamily. Deletion of HCII 1-75 greatly reduces the ability of glycosaminoglycans (GAGs) to accelerate the inhibition of thrombin, and abrogates HCII binding to thrombin exosite 1. While a minor portion of HCII 1-75 can be visualized in a crystallized HCII-thrombin S195A complex, the role of the rest of the extension is not well understood and the affinity of the HCII 1-75 interaction has not been quantitatively characterized. To address these issues, we expressed HCII 1-75 as a small, N-terminally hexahistidine-tagged polypeptide in *E. coli.*

**Results:**

Immobilized purified HCII 1-75 bound active α-thrombin and active-site inhibited FPR-ck- or S195A-thrombin, but not exosite-1-disrupted γ_T_-thrombin, in microtiter plate assays. Biotinylated HCII 1-75 immobilized on streptavidin chips bound α-thrombin and FPR-ck-thrombin with similar K_D_ values of 330-340 nM. HCII 1-75 competed thrombin binding to chip-immobilized HCII 1-75 more effectively than HCII 54-75 but less effectively than the C-terminal dodecapeptide of hirudin (mean K_i_ values of 2.6, 8.5, and 0.29 μM, respectively). This superiority over HCII 54-75 was also demonstrated in plasma clotting assays and in competing the heparin-catalysed inhibition of thrombin by plasma-derived HCII; HCII 1-53 had no effect in either assay. Molecular modelling of HCII 1-75 correctly predicted those portions of the acidic extension that had been previously visualized in crystal structures, and suggested that an α-helix found between residues 26 and 36 stabilizes one found between residues 61-67. The latter region has been previously shown by deletion mutagenesis and crystallography to play a crucial role in the binding of HCII to thrombin exosite 1.

**Conclusions:**

Assuming that the K_D_ value for HCII 1-75 of 330-340 nM faithfully predicts that of this region in intact HCII, and that 1-75 binding to exosite 1 is GAG-dependent, our results support a model in which thrombin first binds to GAGs, followed by HCII addition to the ternary complex and release of HCII 1-75 for exosite 1 binding and serpin mechanism inhibition. They further suggest that, in isolated or transferred form, the entire HCII 1-75 region is required to ensure maximal binding of thrombin exosite 1.

## Background

Heparin cofactor II (HCII) is a 66 kDa plasma protein and member of the serpin superfamily of protease inhibitors [1,2] (reviewed in [3]). It regulates the key coagulation protease thrombin by trapping it in a covalently-linked denaturation-resistant complex. Thrombin-complexed HCII is cleaved at its reactive center peptide bond [4], while thrombin in the inhibitory complex, by analogy to enzymes in other serpin-enzyme complexes, is conformationally inactivated by active site distortions that render it incapable of completing the catalytic cycle of a proteolytic enzyme [5,6]. The rate of inhibition of thrombin by HCII is increased by three to four orders of magnitude by glycosaminoglycans (GAGs) such as heparin, heparan sulfate, or dermatan sulfate [7-11]. The predominant mechanism of GAG acceleration of thrombin inhibition by HCII appears to be allosteric [12-14], although longer GAG chains may have a higher affinity for thrombin than for HCII, introducing some template effects [15]. GAG binding elicits a conformational change in HCII that enables a region of this serpin inhibitor to bind thrombin anion-binding exosite 1, a cluster of charged residues which also engages fibrinogen, thrombomodulin, factor V, and the carboxy-terminal portion of the leech thrombin inhibitor hirudin [16].

Alignment of the primary structure of HCII to other serpins [17] revealed the presence of a unique 75 amino acid N-terminal extension of the protein, one characterized by two repeated negatively charged regions containing 14 glutamic or aspartic acid residues and 2 sulfated tyrosines between positions 42 and 75 of the polypeptide. Deletion of residues 1-67 or 1-74 greatly reduced GAG-catalyzed thrombin inhibition by HCII, while having little effect on progressive, GAG-independent inhibition; deletion of residues 1-52 affected neither mode of inhibition [7]. Charge neutralization of the GAG binding domain in HCII helix D was found to activate HCII for thrombin inhibition in the absence of GAGs [10,11]. Substitution of γ_T_-thrombin, in which exosite 1 has been largely disrupted by proteolysis, for intact α-thrombin in HCII inhibition studies was shown to eliminate most of the GAG-dependent acceleration [7,9,11,18]. These observations were unified in models of HCII inactivation in which GAG binding releases both the N-terminal extension for exosite 1 binding and the reactive centre loop for engagement with the active site of thrombin [7,10,11,18].

Previous work from this laboratory showed that fusion of residues 1-75 of HCII to the N-terminus of another thrombin inhibitory serpin, the M358R “Pittsburgh” variant of α_1_-proteinase inhibitor (α_1_-PI, also known as α_1_-antitrypsin) increased the rate of thrombin inhibition in the absence of GAGs by 21-fold [19]. The rate advantage of the HAPI M358R fusion protein over unfused α_1_-PI M358R was reduced to 6-fold when residues 55-75 were substituted for the entire N-terminal acidic extension, and eliminated altogether either when all acidic residues between 55-75 were neutralized by mutation in the full 1-75 transferred extension, or when residues 1-54 alone were employed for fusion [19]. The rate advantage was also reduced to 9-fold for γ_T_-thrombin versus intact α-thrombin, and was less than 2-fold for inhibition of trypsin or activated protein C [19].

Although HCII has been crystallized both alone and in an encounter complex with S195A-thrombin, the N-terminal acidic extension could not be visualized in the native HCII structure, and was only partially resolved in the HCII-S195A complex, in which residues 56 to 72 were found to form extensive hydrophobic and some electrostatic bonds with thrombin exosite 1 [20]. Although the differences between the encounter complex and the native structure support the allosteric model of activation, they provide no information on the position or role of the first 54 residues of HCII. Moreover, with the exception of one study employing a synthetic peptide to HCII residues 54-75, which showed inhibition of thrombin-mediated fibrinogen cleavage and clot formation [21], there is little information available in the literature addressing the affinity of binding of the HCII N-terminal extension to thrombin. In the current study, we expressed HCII 1-75 as a hexahistidine-tagged small recombinant polypeptide in *E. coli* and examined its binding to various forms of thrombin in comparison to HCII 1-53 and 54-75, as well as to peptides corresponding to the C-terminus of hirudin. We report a higher affinity between thrombin and HCII 1-75 than in the case of HCII 54-75, consistent with our previous findings with α_1_-PI M358R fusion proteins [19]; in addition we report K_D_ values for the HCII 1-75 and thrombin interaction in the 300 nM range.

## Methods

### Peptides

Synthetic peptides corresponding to residues 1-53 and 54-75 of HCII, both preceded by MGSH_6_ sequences with unmodified amino termini, were prepared by the Advanced Protein Technology Centre of the Hospital for Sick Children, Toronto, ON, using 9-fluorenylmethyloxycarbonyl (Fmoc) chemistry and solid phase synthesis. Peptides corresponding to hirudin variant 1 residues 55-65 with an acetylated N-terminus, and residues 54-65 with a free N-terminus and tyrosine sulfation of residue Y63 were purchased from Sigma Aldrich (St. Louis, MO).

### Construction of pBAD-H6-HCII1-75

In order to express HCII 1-75 as an independent polypeptide unconnected to the rest of the HCII protein, plasmid pBAD-H_6_-API M358R [22] was used as the template for PCR, employing sense oligonucleotide 5’-GATCCATGGG GTCTCATCAC CATCACCATC ACGGGAGCAA AGGCCCGCTG GATCAG-3’ [23] and antisense oligonucleotide 5’-GCATGAATTC AGTCGATGTA GTCGTCGTCT TC-3’. The resulting 268 bp amplification product was restricted with NcoI and EcoRI and inserted between the corresponding sites of pBADmychis-B (Invitrogen, La Jolla, CA) to produce pBAD-H_6_-HCII_1-75_. The resulting open reading frame encoded HCII codons 1-75, preceded by nine codons specifying MGSH_6_.

### Construction of pBAD-H_6_HAPI T345R/M358R

In order to express an HCII 1-75-α_1_-PI M358R fusion protein incapable of forming a serpin-enzyme complex with thrombin, the 5153 bp BstXI-EcoRI digestion product of pBAD-H_6_HAPI M358R [19] was combined with the 361 bp fragment formed by digestion of pBAD-API T345R/M358R [24], yielding plasmid pBAD-HAPI T345R/M358R.

### Expression and purification of HCII 1-75

*E. coli* TOP10 cells (Invitrogen, La Jolla, CA) transformed to ampicillin resistance with pBAD-H6-HCII_1-75_ were grown in LB/ampicillin with shaking at 37°C to an OD600 of 0.5, prior to induction with arabinose to 0.002% (w/vol). Following an additional 3.5 hours of growth, cells were harvested by centrifugation and cell pellets disrupted by sonication in equilibration buffer (EB; 50 mM sodium phosphate pH 8.0, 300 mM NaCl, 10 mM imidazole), then made 1% (vol/vol) in Triton X-100. The clarified lysate was applied to nickel-nitrilotriacetic acid (Ni-NTA) agarose resin (Qiagen, Carlsbad, CA), washed, and eluted with EB containing 250 mM imidazole. Peak fractions were dialyzed versus 20 mM Tris-Cl pH7.4, 200 mM NaCl, and then chromatographed on Q-Sepharose (GE Healthcare, Baie d’Urfe, QC). Proteins remaining bound after washes with 20 mM Tris-Cl pH7.4, 300 mM NaCl were eluted with 20 mM Tris-Cl pH7.4, 350 mM NaCl and concentrated with an Amicon Ultra-15 Centrifugal Filter Unit with Ultracel-3 membrane (EMD Millipore, Billerica, MA). The concentration of purified HCII 1-75 (and of all other purified peptides and proteins employed in this study) was determined by measuring the OD280 using a spectrophotometer and the method of Edelhoch to estimate the molar extinction coefficient based on the primary sequence [25,26], which yielded a value of 33920 M^-1^cm^-1^.

### Mass spectrometry

The mass of purified HCII 1-75 in a solution of 10 mM Tris-Cl pH 7.4 was determined, using Matrix-Assisted Laser Desorption Ionization Mass Spectrometry (MALDI MS) by the Advanced Protein Technology Centre of the Hospital for Sick Children (Toronto, ON).

### Expression and purification of HCII-α_1_-PI fusion proteins

His-tagged recombinant proteins were purified from cell lysates prepared from arabinose-induced *E. coli* TOP10 cells transformed to ampicillin resistance, either with previously described plasmid pBAD-H_6_HAPI M358R or with the novel construct pBAD-H_6_HAPI T345R/M358R; the same protocol was employed, involving Ni-NTA agarose (Qiagen) and DEAE-Sepharose (GE Healthcare, Baie d’Urfe, QC) chromatography of lysates from sonically disrupted bacteria as previously described [19].

### Preparation of S195A-thrombin

S195A-thrombin was produced from purified prothrombin-1 S195A expressed in Baby Hamster Kidney Cells (the generous gift of Dr. Timothy Mather, Oklahoma City, OK), as previously described, using bovine prothrombinase digestion and SP-Sepharose (GE Healthcare) chromatography [27,28].

### Gel-based analysis of serpin-enzyme complexes

Formation of HAPI-thrombin complexes was followed by incubating 1 μM HAPI M358R or HAPI T345R/M358R with 200 nM α**-**thrombin (Enzyme Research, South Bend, IN) at 37°C for 1 minute prior to stopping the reaction with one volume of 2X SDS-PAGE Loading Buffer (0.16 M Tris-Cl pH 6.8, 4.4% (vol/vol) SDS, 20% vol/vol glycerol, 0.2 M dithiothreitol, 0.4 mg/ml Bromophenol Blue) and visualizing reaction products following SDS-PAGE as described [22,29,30].

### Thrombin binding assay

**Figure 1:**
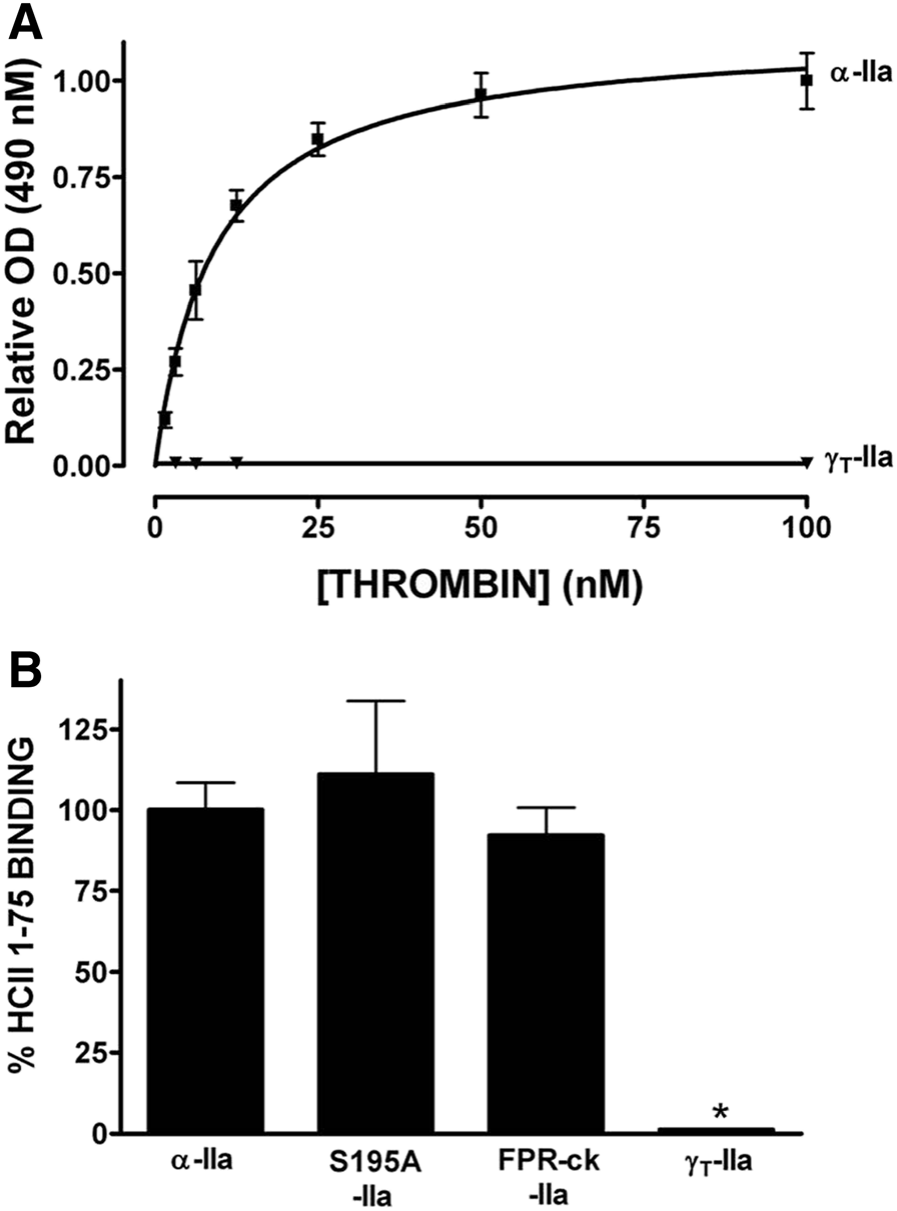
**Binding of different forms of thrombin to HCII 1-75 immobilized on microtiter plate wells.** Binding of α-thrombin (α-IIa, solid squares) or γT-thrombin (γT-IIa, ▾) was measured as the optical density (OD) at 490 nm as described in Materials and Methods, and graphed relative to the mean OD of binding reactions carried out with 100 nM α-thrombin, taken as 100%, in Panel **A**. The mean ± the SD of 3 independent experiments is shown. Panel **B** shows the results of experiments similar to those shown in Panel **A**, but carried out with 12.5 nM α-thrombin (α-IIa), S195A-thrombin (S195A-IIa), FPR-ck-thrombin (FPR-ck-IIa) or γT-thrombin (γT-IIa); results were normalized to the mean optical density (OD) at 490 nm observed for α-thrombin binding. Each bar represents the mean ± the SD of 3 independent experiments; * indicates p<0.05 by non-parametric ANOVA with Dunn’s post-test versus α-thrombin binding.

**Figure 2:**
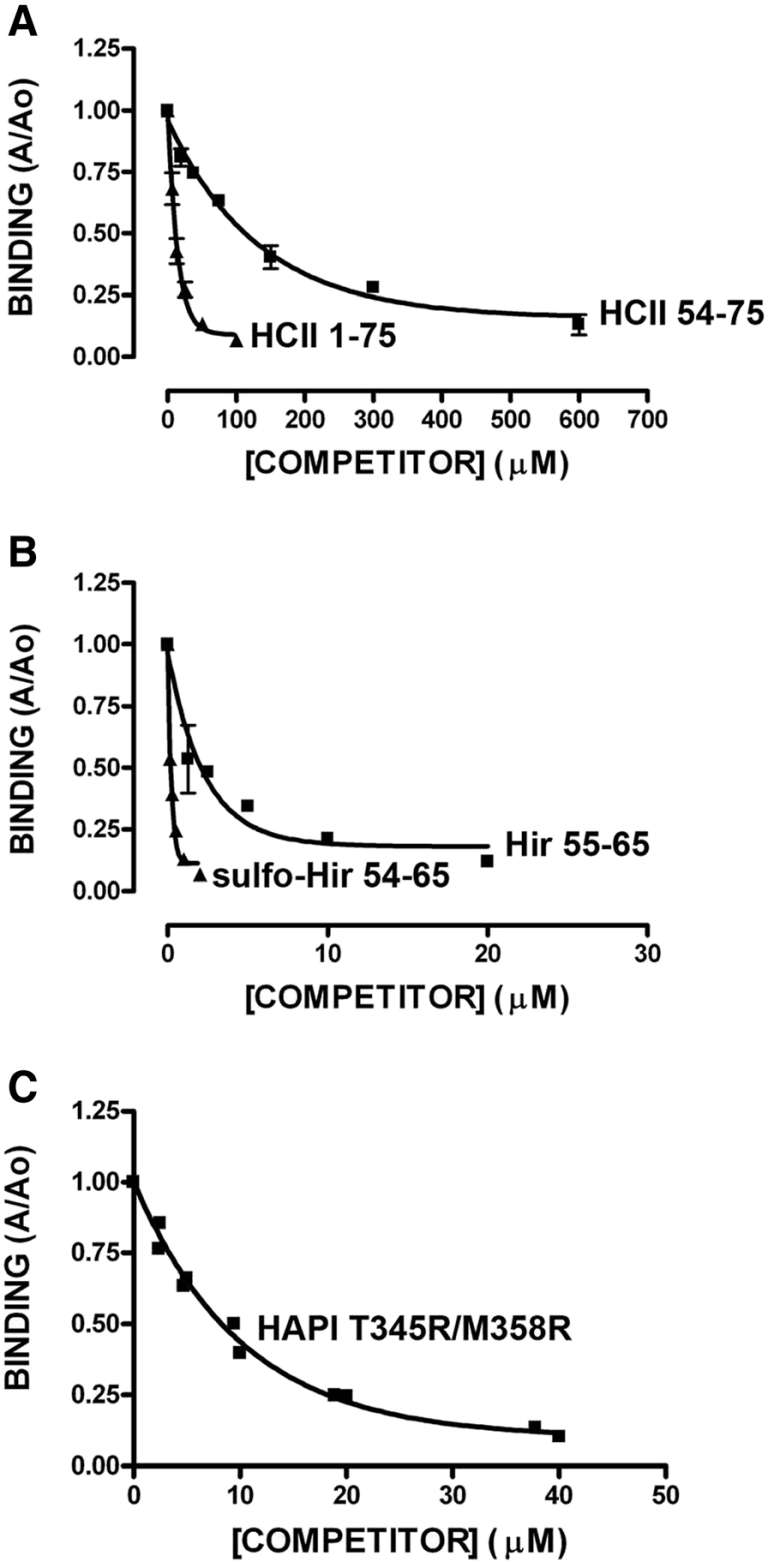
**Competition of binding of α-thrombin to HCII 1-75 immobilized on microtiter plate wells.** Binding of α-thrombin was measured as the absorbance at 490 nm as described in Figure 1, and expressed as the ratio of the absorbance in the presence of competitor (A) to that in its absence (Ao). Competitor peptides identified to the right (panels **A** and **B**) or above (panel **C**) the competition curve. The mean ± the SD of 3 independent experiments is shown.

Purified HCII 1-75 was immobilized onto an Immulon 4 HBX 96 well microtiter plate (Thermo, Milford MA), using a 1 μM solution of HCII 1-75 in PBS at 0.1 mL per well overnight at 4°C. The HCII solution was then removed and the wells blocked with 5% BSA (w/vol) in PBS-T for one hour at ambient temperature. Following washes with PBS-T, 0-100 nM α-thrombin in blocking buffer was added to the wells and incubated at 37°C for 30 minutes. Wells were washed and reacted with horseradish peroxidase-conjugated sheep anti-human thrombin antibody (Affinity Biologicals, Ancaster, ON), diluted 1:10,000 in blocking buffer, for one hour at ambient temperature. Following washes with PBS-T, colour was developed using a freshly made solution of 5 mg o-phenylenediamine (Pierce) in 12 mL of 25 mM citric acid, 97 mM Na_2_HPO_4_ pH5.0 supplemented with 0.03% (v/vol) hydrogen peroxide for ten minutes prior to stopping the reaction with sulfuric acid. The optical density at 490 nm was then read on an EL808 Plate reader (Biotek Instruments, Winooski, VT). In some experiments, competitor peptides were added with the thrombin; in others S195A-thrombin or thrombin inactivated at its active site using D-Phe-L-Pro-L-Arg chloromethylketone (FPR-ck, EMD Millipore, Billerica, MA) was substituted for α-thrombin. Binding isotherms such as those shown in Figure 1A were curve-fit for one-site binding (hyperbola) using non-linear regression in GraphPad Prism 4.0 software (GraphPad Software, La Jolla, CA). The same program was used to calculate IC50s in competitive binding experiments in which HCII-derived or hirudin-derived peptides were used to compete the binding of thrombin to immobilized HCII 1-75. Inhibition plots such as those shown in Figure 2 were logarithmically transformed, lines of best fit solved by linear regression, and the competitor concentration reducing binding to 50% (IC_50_) was determined.

### Thrombin clotting times

Thrombin times were determined using Thrombin 10 (freeze-dried human thrombin with calcium [1.5 NIH units/mL] Diagnostica Stago, Asnieres, France) and an STA 4 coagulation analyzer (Diagnostica Stago). Citrated human plasma (25 μL) was combined with 75 μL veronal buffer (7.14 mM sodium acetate trihydrate, 7.4 mM sodium diethyl barbiturate, 131 mM NaCl pH 7.4) containing or lacking HCII- or hirudin-related peptides, and clotting time was determined following the addition of 100 μL Thrombin 10 reagent.

### Biotinylation of peptides and proteins

HCII- or hirudin-related peptides in 50 mM sodium phosphate pH 6.5 were combined with 20-fold molar excess sulfosuccinimidyl-6-(biotinamido) hexanoate (sulfo-NHS-LC-biotin, Pierce) at ambient temperature for 30 minutes. Reactions were quenched with excess Tris-Cl pH 7.4 and sulfo-NHS-LC-biotin removed by desalting or dialysis. Biotinylated FPR chloromethylketone (bFPRck; Haematologic Technologies, Essex Junction, VT) was used to modify α-thrombin at a bFPRck: thrombin molar ratio of 10:1 in Tris-buffered saline (TBS, pH 7.4) for ten minutes at ambient temperature. Excess reagent was removed by overnight dialysis versus TBS. Thrombin biotinylated by bFPRck had no detectable amidolytic activity against S2238.

### Surface plasmon resonance

Surface plasmon resonance experiments were carried out using a BIAcore X biosensor instrument (Biacore, Piscataway, NJ) at 25°C. Biotinylated HCII- or hirudin-related peptides were immobilized on streptavidin-coated sensor chips (GE Healthcare) under conditions recommended by the manufacturer. One flow cell contained bound peptide and the other did not, serving as a reference cell to correct for background binding effects. A flow rate of 2 μL/minute was employed for immobilization runs, with 0.5 M NaCl employed as a regeneration buffer and HEPES-buffered saline (10 mM HEPES, 150 mM NaCl, 0.005% Tween-20, pH 7.7) otherwise used for binding determinations. Binding of thrombin (0-30 μM) was assessed using 20 μL injection volumes, and flow rates of 20 μL/minute. BIAcore Evaluation 3.2 (BIAcore) software was used to evaluate sensorgrams from each run, using either 1:1 Langmuir binding or steady state analysis. In some experiments various concentrations of HCII- or hirudin-related peptides were combined with 250 nM thrombin or FPR-ck-thrombin immediately prior to being flowed over chip-bound HCII 1-75.

### Rate determinations for thrombin inhibition by plasma-derived HCII

The apparent second-order rate constant (k_2_app) of α-thrombin inhibition by purified plasma-derived HCII (Affinity Biologicals, Ancaster, ON) was determined under pseudo-first order conditions involving an excess of plasma-derived HCII over α-thrombin using a discontinuous assay as previously described [19,30], with or without addition of competitor HCII 1-75, HCII 54-75, or HCII 1-53 at 0.25 mM. Briefly, plasma-derived HCII (140 nM) was incubated with α-thrombin (14 nM) in PPNE buffer (20 mM Na_2_HPO_4_, pH 7.4, 100 mM NaCl, 0.1 mM EDTA, 0.1% polyethylene glycol 8000) supplemented with 0.055 mg/mL (2 μM) standard heparin (Hepalean, Organon Teknika Canada, St. Laurent, QC) and 10 μM S2238 (DiaPharma,West Chester OH) or 0.055 mg/mL (1.4 μM) dermatan sulfate (Sigma-Aldrich, St. Louis, MO). Residual protease activity was determined by diluting the reactions 100-fold into 100 μM S2238 and measuring the absorbance at 405 nm for 5 minutes using an EL808 plate reader (BioTek Instruments, Winooski, VT). From these data, pseudo first-order and second-order rate constants were derived as described, correcting for the presence of chromogenic substrate in the first stage of the reaction [30].

### Molecular modelling of HCII 1-75

Molecular modeling of the first 75 amino residues of human HCII was performed using 3D-Jigsaw Comparative Modeling software [31] in the automatic mode. This software makes use of a threading algorithm dependent on secondary structure prediction by PSIPRED [32]. The predicted HCII1-75 molecular model fell within the 95th percentile for expected structural accuracy (E value of 5.0 x 10^-5^). Structural superimposition was performed with COOT [33]. Images were prepared with PyMol (The PyMOL Molecular Graphics System, Version 1.2, Schrödinger, LLC).

## Results

### Expression and initial characterization of recombinant HCII 1-75

**Figure 3:**
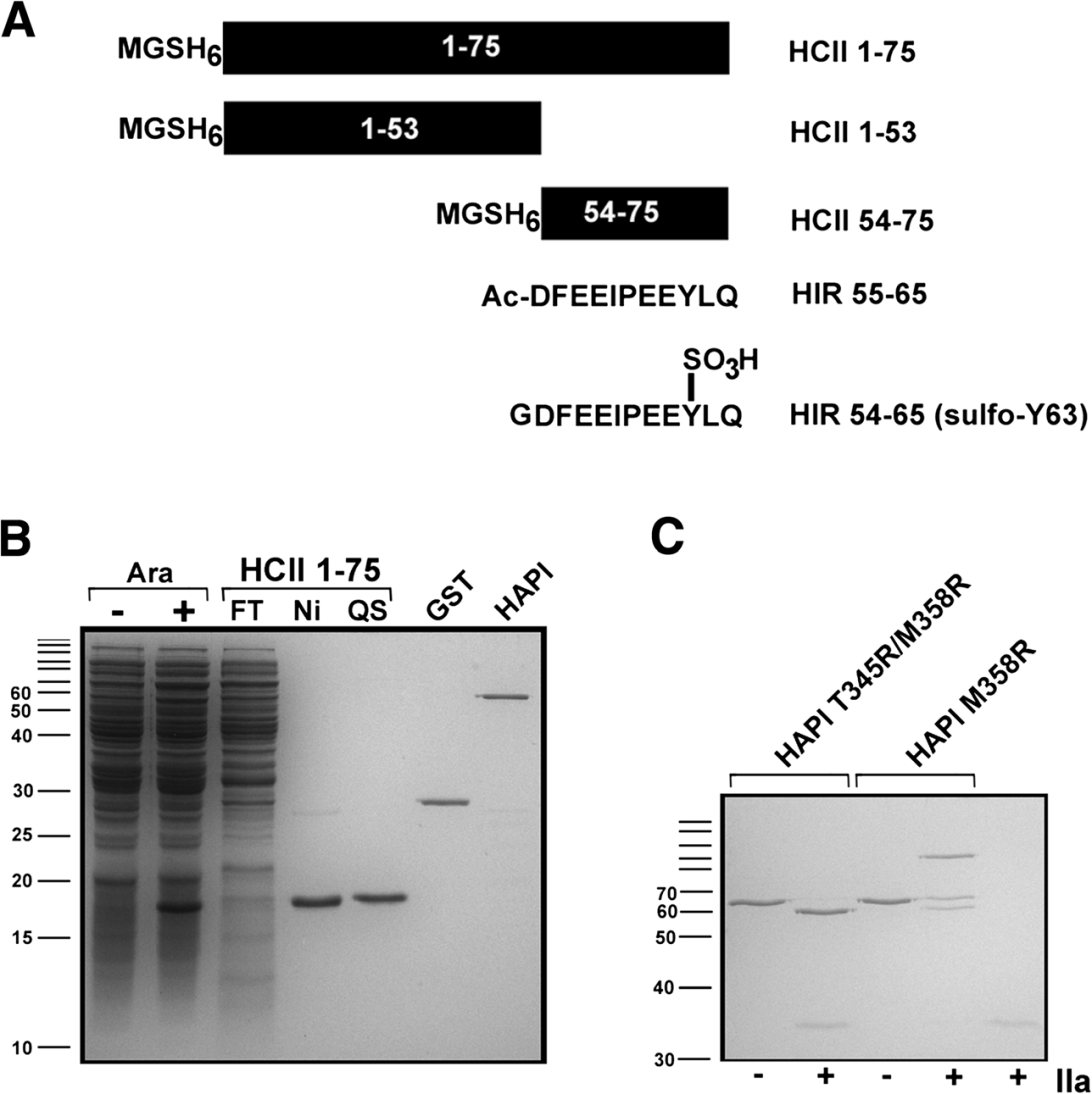
**Schematic and electrophoretic representation of proteins and peptides employed in this study.** Polypeptides comprising portions of HCII (solid black bar, with residue numbers inset in white) or hirudin (Hir) (primary sequence shown) are represented schematically in panel **A**. Each HCII-derived protein or peptide contains an N-terminal MGSH_6_ tag (shown at left of each schematic representation): Ac, acetyl; SO_3_H, sulfate group on Hir Tyr63. Panel **B** shows a reduced 12% SDS polyacrylamide gel stained with Coomassie Brilliant Blue. Ara- and Ara+ lanes show total bacterial lysates from cultures expressing HCII 1-75 grown in the presence or absence of 0.002% (w/vol) arabinose. Aliquots of bacterial lysates purified by nickel chelate chromatography (FT, flow-through; Ni, imidazole-eluted peak fractions; QS, final preparation polished by ion exchange on Q-Sepharose) are shown. Approximately 0.75 μg of purified HCII 1-75 (QS), glutathione sulfotransferase (GST) and HAPI M358R (HCII 1-75 α_1_-PI M358R fusion protein) were electrophoresed in the last three lanes at right. Panel **C** shows the reaction products of purified HAPI M358R or HAPI T345R/M358R incubated with (+) or without (-) thrombin (IIa) electrophoresed on a reduced 12% SDS polyacrylamide gel stained with Coomassie Brilliant Blue. The positions of molecular mass markers are labeled, in kDa, to the left of panels **B** and **C**; positions that are not labeled due to insufficient space correspond to 160, 120, 100, 90, and 80 kDa.

We chose to express HCII 1-75 as a small recombinant protein in *E. coli* because several laboratories have shown that HCII 1-75 can fold appropriately when expressed in *E. coli* in the context of the full HCII polypeptide [7,30,34,35]. Moreover, this laboratory has shown that HCII 1-75 retains the ability to interact with thrombin when expressed in *E. coli* as part of HCII 1-75 - α_1_-PI M358R fusion proteins [19]. As shown in Figure 3A, the same sequence that was selected for use in the full-length proteins was employed, such that an initiator Met codon was followed by Gly-Ser codons introduced in the DNA manipulations, 6 His codons, and the N-terminal 75 codons of HCII. Inspection of total cellular protein profiles from *E. coli* harbouring plasmids encoding HCII 1-75 on Coomassie Blue-stained SDS PAGE gels revealed an arabinose-inducible polypeptide of approximately 16 kDa (see Figure 3B, Ara- vs Ara+ lanes). This polypeptide bound to and eluted from nickel chelate columns (Figure 3B); purification to homogeneity was achieved following polishing by ion exchange (Figure 3B, Q Sepharose (QS) peak). Purified HCII 1-75 reacted both with anti-hexahistidine and anti-HCII antibodies on ELISA (data not shown). Because the mobility of this putative HCII 1-75 polypeptide differed from its theoretical full-length molecular weight of 9624 Da, confirmation of its mass by spectrometry was undertaken. MALDI mass spectrometry revealed a single major peak corresponding to a mass/charge ratio of 9494; this value is within one atomic mass unit of the theoretical mass of HCII 1-75 lacking its initial Met residue (9493) (data not shown). Taken together, these observations confirmed the identity of the polypeptide as recombinant HCII 1-75.

### Expression of recombinant HCII 54-75 and synthesis of HCII 54-75 and 1-53

Expression of recombinant HCII 54-75 was attempted using an analogous approach to that employed for HCII 1-75. However, no arabinose-inducible proteins were identified, either via SDS gel or immunoblot analysis of either bacterial lysates or nickel chelate-selected proteins (data not shown). We therefore obtained HCII 54-75 and HCII 1-53 as synthetic peptides produced using custom solid phase synthesis, as well as commercial synthetic peptides corresponding to the acetylated C-terminal 11 residues of hirudin variant 1 (Hir 55-65) or the tyrosine-sulfated C-terminal dodecapeptide of hirudin variant 1 (sulfo-Hir 54-65) as shown schematically in Figure 3A.

### Elimination of serpin activity of HAPI T345R/M358R

Fusion protein HAPI M358R contains the same sequence as recombinant HCII 1-75 employed in this study, but fused to the N-terminus of α_1_-PI M358R via a hexaglycine spacer (19). In order to produce a variant of this protein incapable of forming a covalent serpin-enzyme complex with thrombin, in which interaction with thrombin could occur only via the N-terminal HCII extension, we incorporated an additional T345R mutation into the protein, one previously shown to inactivate α_1_-PI [36] and cell-surface tethered α_1_-PI M358R [37]. As shown in Figure 3C, reaction of purified HAPI M358R with thrombin led to the formation of both a 100 kDa serpin-thrombin denaturation-stable complex, and a 61 kDa cleaved form of the recombinant serpin; in contrast, the identical reaction carried out with HAPI T345R/M358R led to the generation of cleaved serpin alone.

### Binding of different forms of thrombin to immobilized HCII 1-75

An assay was developed to assess the binding of thrombin to HCII 1-75 immobilized on microtiter plate wells, using an antibody to thrombin and enzymatic colour generation linked to the antibody. In order to compare the results of different experiments, results (shown in Figure 1A) were normalized to the mean optical density elicited by 100 nM concentrations of α-thrombin in all replicated experiments. Binding was saturable (note the lack of difference between binding elicited by 50 and 100 nM α-thrombin) and specific, since substitution of γ_T_-thrombin for α-thrombin eliminated binding (Figure 1A, lower curve). γ_T_-thrombin is a proteolyzed derivative of α-thrombin in which most of thrombin exosite 1 has been eliminated [9]. When the thrombin concentration was fixed at 12.5 nM, below saturation on the binding curve (Figure 1A), and binding of different forms of thrombin was compared to that of α-thrombin, no difference in binding for S195A-thrombin or FRP-ck-thrombin was noted; however, binding was significantly reduced, to minimal levels, using γ_T_-thrombin (Figure 1B). We did not further analyse the binding from this assay, for instance in terms of calculating a dissociation constant, because we had to discard the unbound thrombin and add an antibody to determine the degree of binding, potentially changing the initial binding isotherm. Nevertheless, our results suggested that the assay reported specific binding of HCII 1-75 to thrombin exosite 1 and could be useful for initial experiments.

### Competition for binding of thrombin to immobilized HCII 1-75

We next compared the ability of different exosite 1 ligands to compete for binding of thrombin to HCII 1-75 immobilized on microtiter plate wells. Figure 2 shows the results of these binding experiments, expressed as the ratio of binding in the presence of competitor to binding in its absence. Inspection of the competitive binding curves shows that HCII 1-75 more effectively competed its own binding to 5 nM thrombin than did HCII 54-75 (Figure 2A), while sulfo-Hir 54-65 more effectively competed that binding than Hir 55-65; HAPI T345R/M358R closely resembled HCII 1-75 as a competitor (Figure 2C). The competitive binding curves were linearized by logarithmic transformation and the IC_50_s were calculated for replicated experiments (see Methods). The IC_50_s of the competitors, in order of decreasing affinity (in μM, mean of 3 determinations ± SD), were found to be: sulfo-Hir 54-65 (0.140 ± 0.040); Hir 55-65 (2.40 ± 0.30); HAPI T345R/M358R (8.24 ± 0.33); HCII 1-75 (11.0 ± 4.0); and HCII 54-75 (110 ± 20).

### Prolongation of thrombin clotting time by HCII and hirudin peptides

**Figure 4:**
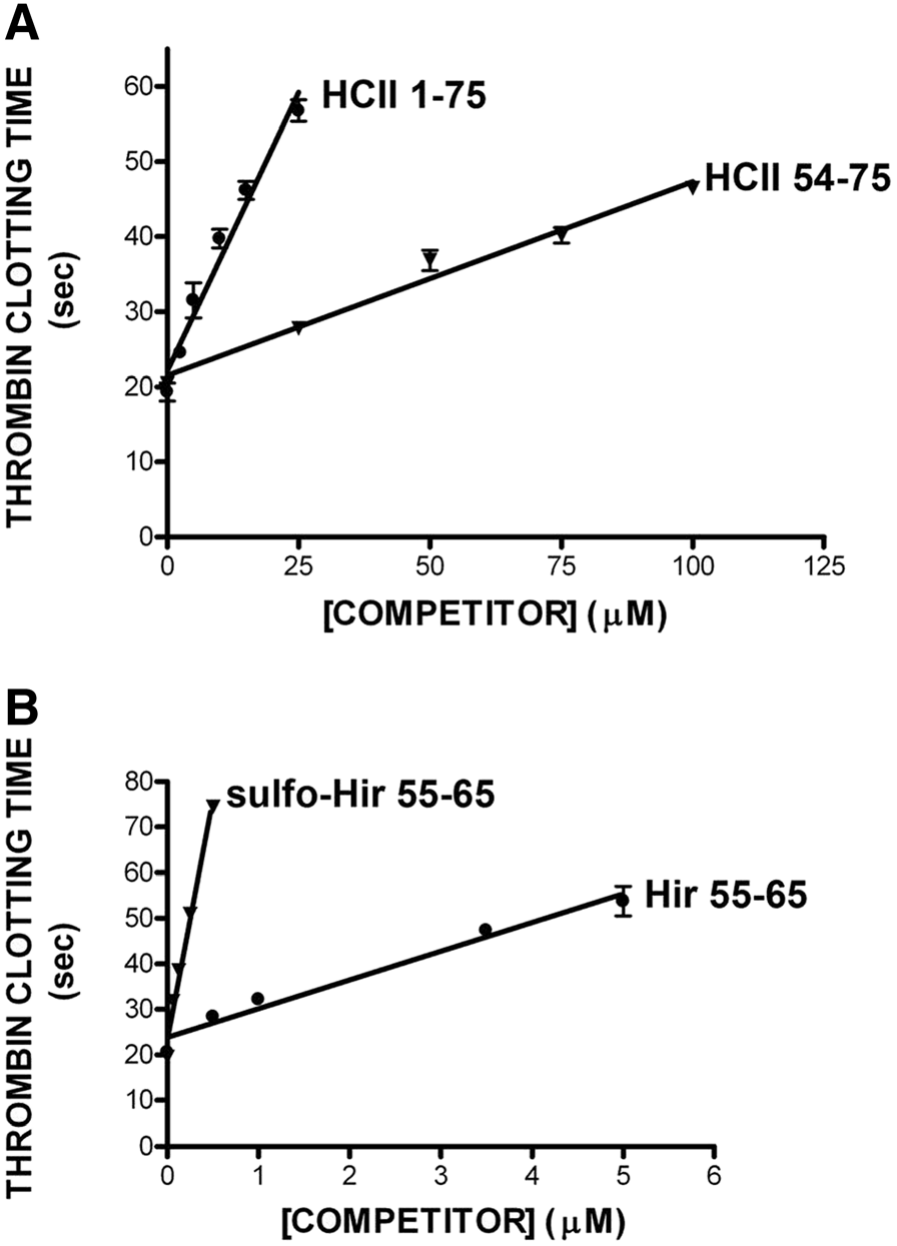
**Effects of competitor peptides on thrombin clotting time.** Diluted citrated human plasma was recalcified in the presence of thrombin and increasing concentrations of competitor peptides identified to the right of each line (either HCII 1-75 or HCII 54-75 (panel **A**) or sulfo-Hir 54-65 or Hir 55-65 (panel **B**)). The mean ± the SD of 3 independent experiments is shown.

An HCII 54-75 peptide had previously been shown to be approximately 30-fold less potent than sulfo-Hir 54-65 as an inhibitor of thrombin-mediated clotting of recalcified human plasma [21]. We replicated this experiment and extended it to include all four of the exosite 1 ligand peptides employed in this study, as shown in Figure 4. We used the measurement previously employed to judge the potency of the peptides, the concentration required to double the thrombin clotting time [21]. The following order of efficacy as inhibitors of thrombin in plasma was observed (in μM, mean of 3 determinations ± SD): sulfo-Hir 54-65 (0.150 ± 0.02); Hir 55-65 (2.60 ± 0.40); HCII 1-75 (13.0 ± 1.7) and HCII 54-75 (72.0 ± 6.4).

### Thrombin inhibition by plasma-derived HCII in the presence of heparin and HCII peptides

**Figure 5:**
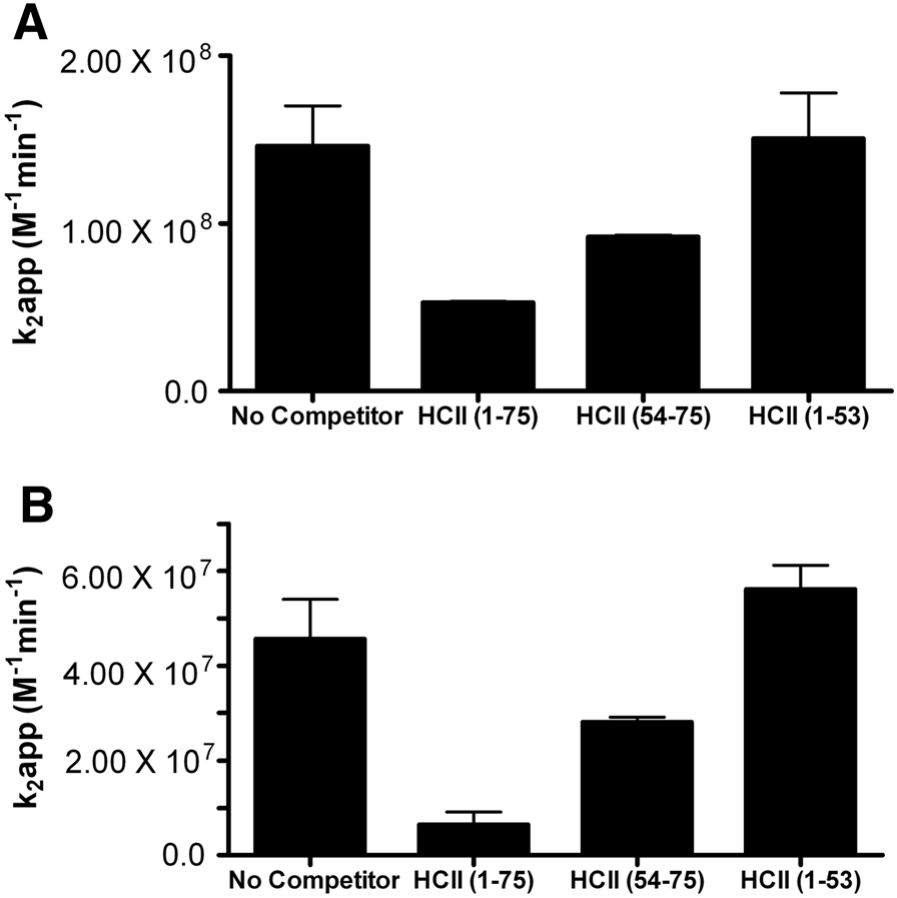
**Effects of HCII-derived peptides on the inhibition of thrombin by plasma-derived HCII in the presence of GAGs.** The second-order rate constant of inhibition (k2) for the inhibition of 14 nM thrombin by 140 nM plasma-derived HCII was determined in the presence of 55 μg/mL standard heparin (panel **A**) or dermatan sulfate (panel **B**) in the absence (No Competitor) or presence of competing peptides. The competing peptides that were used separately at 250 μM were: HCII 1-75; HCII 54-75; or HCII 1-53. The mean ± the SD of 3 independent experiments is shown.

To compare the ability of the entire N-terminal acidic extension of HCII to its constituent parts to compete for plasma-derived HCII in inhibiting thrombin, the second order rate constant of inhibition was determined in the presence of heparin or dermatan sulfate, in a two-stage reaction. The apparent reduction in this constant was then determined, as shown in Figure 5A; when 250 μM competitor peptide was added to 140 nM plasma-derived HCII in the presence of 55 μg/mL standard heparin or dermatan sulfate, HCII 1-53 had no effect, while HCII 1-75 reduced the apparent rate of inhibition to a greater extent than HCII 54-75 (on average by 2.8-fold versus 1.6-fold). The same pattern was seen in analogous reactions in which 1.4 μM dermatan sulfate was employed (Figure 5B), in that no reduction in the apparent rate of inhibition was elicited by HCII 1-53, but HCII 1-75 reduced it 7.0-fold and HCII 54-75 only 1.6-fold.

### Surface plasmon resonance

**Figure 6:**
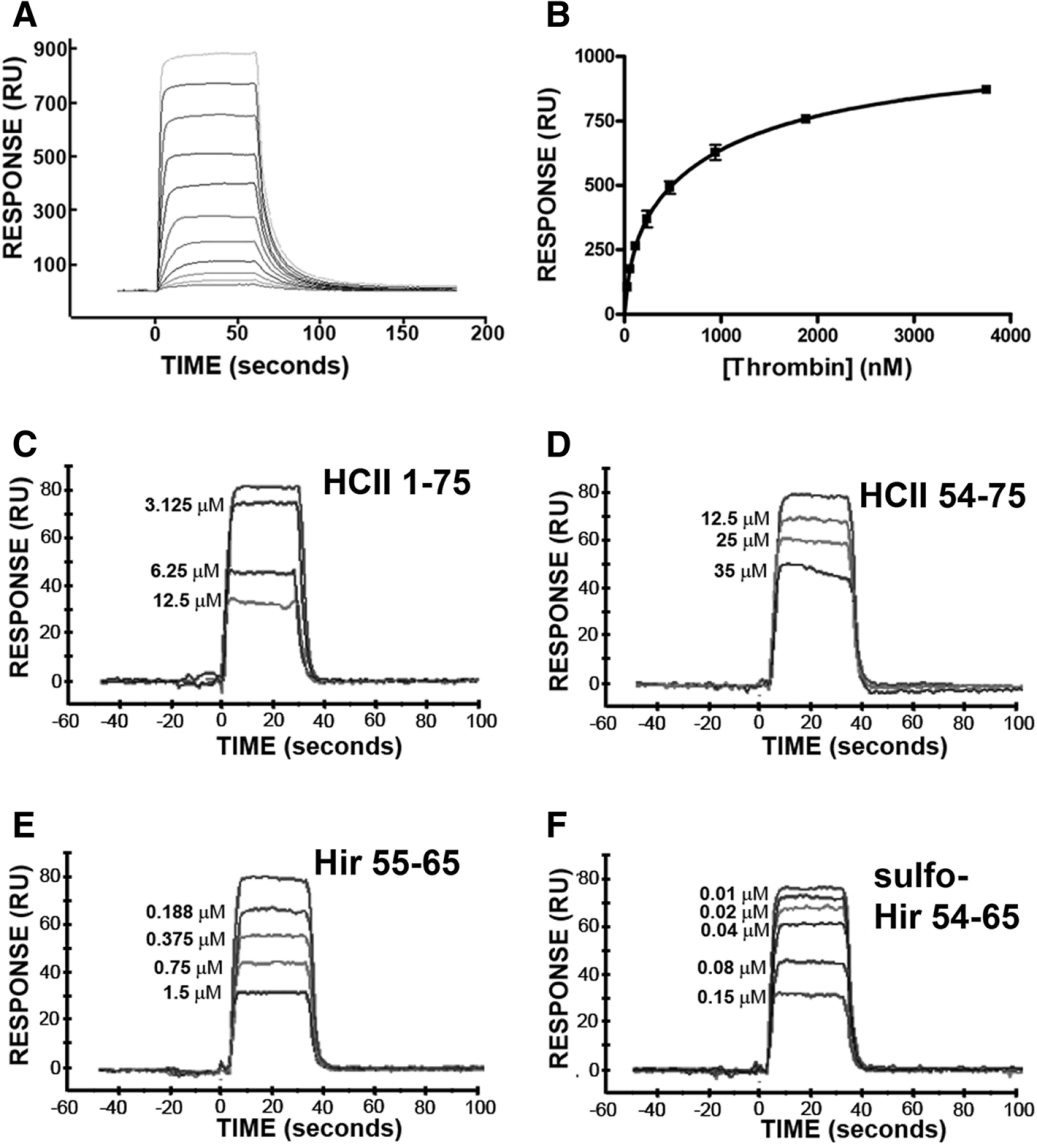
**Senorgrams from surface plasmon resonance (SPR) experiments.** Biotinylated HCII 1-75 was bound to streptavidin-coated sensor chips and varying concentrations of thrombin were flowed over the chip, generating sensorgrams (e.g. Panel **A**) of the response in response units (RU) versus the time in seconds. Panel **B** is a plot of experiment shown in Panel **A**, combined with two other independent experiments; each point therefore represents the mean mid-plateau response ± the SD of 3 independent experiments. In panels **C-F** the thrombin concentration was fixed at 250 nM and competitor peptides identified in each panel were combined with thrombin and flowed over the chip. The concentration of competitor peptides in each experiment is shown to the left of the sensorgram.

In order to facilitate immobilization on sensor chips for surface plasmon resonance (SPR), HCII 1-75 was biotinylated under conditions reported to favour modification of its N-terminus, as opposed to amine-containing side chains [38]. This modification did not appear to affect the ability of the polypeptide to inhibit thrombin, since 11.7 μM biotinylated HCII 1-75 was required to double the thrombin clotting time, a concentration not different from that of the unmodified polypeptide (12.8 ± 1.0 μM) (data not shown). Biotinylated HCII 1-75 was immobilized on streptavidin-coated sensor chips, and binding resulting from flowing different concentrations of thrombin over the chip was observed by SPR as shown in the sensorgrams in Figure 6A. Plotting the results of three independent experiments and fitting the data to a one site binding model yielded a K_D_ of 340 ± 40 nM (mean ± SD). Substitution of FPR-ck-thrombin for α-thrombin in analogous SPR experiments gave similar K_D_ values of 330 ± 60 nM (data not shown).

### Competition of thrombin binding to chip-immobilized HCII 1-75

Having established thrombin binding conditions appropriate for quantitative analysis using chip-immobilized HCII 1-75, we next assessed the ability of various peptides to compete for that binding. Sensorgrams generated in the presence of increasing concentrations of HCII 1-75, HCII 54-75, Hir 55-65 and sulfo-Hir 54-65 showed decreasing equilibrium binding of 250 nM thrombin, as shown in Figure 6C through 6F. In contrast, addition of HCII 1-53 at concentrations up to 250 μM had no effect on thrombin binding to HCII 1-75 (data not shown). Using the mean K_D_ of 340 nM determined above for α-thrombin and HCII 1-75 binding, and IC_50_s determined by linear regression of the plotted ratios of binding in the presence and absence of the different competitors, constants of inhibition (K_i_) values were determined. As shown in Table 1, HCII 54-75 was less effective at competing thrombin binding than HCII 1-75, as indicated by its 3.3-fold higher mean K_i_. Combining equimolar HCII 54-75 and HCII 1-53 failed to recreate the efficacy of HCII 1-75 as a competitor; the combination was 1.5-fold less effective than HCII 54-75 in inhibiting thrombin binding. Both hirudin-derived peptides were more effective competitors of thrombin binding than HCII 1-75, by 9-fold (Hir 55-65) and 48-fold (sulfo-Hir 54-65), respectively.

**Table 1:** Dissociation constants for inhibition of thrombin binding to HCII 1-75 (SPR)

**Inhibitor**	**K**_**i **_**(μM)**
HCII 1-75	2.6 ± 0.6
HCII 54-75	8.5 ± 2
HCII 1-53	No inhibition
HCII 54-75 and HCII 1-53	13 ± 3
Hir 55-65	0.29 ± 0.07
sulfo-Hir 54-65	0.054 ± 0.09

The mean of 3 determinations ± the standard deviation (SD) is shown.

## Discussion

Although multiple lines of evidence suggest that HCII becomes conformationally activated by GAG binding, leading to the release of the HCII 1-75 N-terminal acidic extension and binding of thrombin exosite 1 [10-14,20], little information was available concerning the affinity of that binding prior to this study. This paucity of data likely arose due to the difficulty of studying the interaction using either plasma-derived or recombinant HCII molecules, which are capable both of forming non-covalent links between HCII 1-75 and thrombin exosite 1 in the presence of GAGs, and a covalent complex of the serpin type with thrombin’s active site. To better understand the role of the HCII acidic extension as a ligand of thrombin exosite 1, we first truncated recombinant His-tagged HCII at residue 75. We employed the same *E. coli* expression system we previously used to express HCII 1-75 fused to α_1_-PI M358R, because HCII 1-75 in that context appeared to bind thrombin exosite 1 [19].

Although purified recombinant MGSH_6_-tagged HCII 1-75 migrated less rapidly than expected on SDS-PAGE, it displayed the expected mass by spectrometry; its atypical migration was likely due to its binding less SDS per molecule than most proteins, as previously noted for fusion proteins containing MGSH_6_-tagged HCII 1-75 [19]. Our failure to express either MGSH_6_-tagged HCII 54-75 or 1-53 in *E. coli* suggested that HCII 1-75 was near the lower size limit for efficient bacterial expression of heterologous polypeptides [39]. We nevertheless ensured appropriate comparisons by including MGSH_6_ in all three HCII derivatives, whether obtained by bacterial expression or chemical synthesis.

Several lines of evidence suggested that our novel, bacterially expressed HCII 1-75 protein functioned as a thrombin exosite 1 ligand. Firstly, it bound intact or active-site modified thrombin, but not exosite 1-diminished γ_T_-thrombin, when immobilized either on microtiter plates or sensor chips. Secondly, this binding was inhibited by peptides whose ability to bind exosite 1 has been validated by co-crystallization with thrombin (sulfo-Hir 54-65 [40] or HCII 54-75 in intact HCII [20]). Thirdly, like HCII 54-75 or sulfo-Hir 54-65 peptides previously synthesized by other investigators [8,21,41], it inhibited thrombin-induced plasma clot formation. Finally, like HCII 54-75, it reduced the apparent rate of thrombin inhibition by plasma-derived HCII in the presence of GAGs. Taken together, our data suggest that HCII 1-75 functioned as an exosite 1 ligand and therefore resembled its native counterpart in intact HCII, once the N-terminal extension had been liberated from intramolecular interactions with the body of HCII by GAG binding. HCII 1-75 understandably best represents its native counterpart in recombinant HCII made in bacterial systems, rather than plasma-derived HCII, because of the inability of bacteria to carry out the tyrosine sulfation of Tyr60 and Tyr73 found in the natural protein [21]. Bacterially expressed forms of HCII nevertheless exhibit similar maximal rates of thrombin inhibition to plasma-derived HCII [7,29].

We employed surface plasmon resonance to determine the K_D_ of HCII 1-75 binding to thrombin, after confirming that biotinylation of HCII 1-75 did not interfere with its ability to delay the thrombin-induced clotting of plasma, and binding the biotinylated HCII 1-75 to streptavidin-coated sensor chips. Flowing either intact α-thrombin or FPR-ck-thrombin over immobilized HCII 1-75 yielded indistinguishable values of 330 or 340 nM for the K_D_, as expected if binding was mediated by exosite 1. While there are no similar values in the literature available for HCII 1-75 for comparison to our results, a 96 nM K_D_ has been reported for another thrombin exosite 1 ligand, the C-terminal hirudin derivative sulfo-Hir 53-64 [42] (also known as hirugen) [43]. Competition of thrombin binding to chip-immobilized HCII 1-75 using either HCII 1-75 itself (K_i_ 2.6 μM) or non-sulfated Hir 55-65 (K_i_ 0.29 μM) or sulfo-Hir 54-65 (K_i_ 0.054μM) confirmed both the superiority of the hirudin derivatives and the contribution of tyrosine sulfation to their exosite 1 binding. Although producing HCII 1-75 in *E. coli* prevented its tyrosine sulfation, non-sulfated Hir 55-65 clearly bound exosite 1 with higher affinity than HCII 1-75, showing that the superiority of the hirudin-derived peptides was manifested by their polypeptide composition alone. Our data are consistent with the previous finding that non-sulfated HCII 54-75 was 30-fold less potent than hirudin-derived, non-sulfated peptides in either thrombin binding or clotting assays [21].

As part of our dissection of the HCII acidic tail, we compared HCII 1-75 to HCII 1-53 and HCII 54-75. HCII 1-53 had no detectable ability to bind thrombin, to delay thrombin-mediated coagulation, or to compete for the N-terminal HCII 1-75 extension in native HCII, mobilized for exosite 1 binding by GAG activation. In contrast, HCII 54-75 had detectable activity in all of the above respects; however, in each setting, it displayed lesser inhibitory potency than HCII 1-75, consistent with its lesser ability to compete for thrombin binding to chip-immobilized HCII 1-75 than free HCII 1-75 (compare K_i_ values of 2.6 to 8.5 μM). If HCII 1-53 does not bind to thrombin directly, then the superiority of HCII 1-75 over HCII 54-75 would seem most likely to derive from a conformational effect on residues 54-75 that increases their ability to bind thrombin in the way visualized in the S195A-thrombin-HCII structure [20]. Combining HCII 1-53 and HCII 54-75 in competitive binding SPR experiments did not improve the ability of the latter peptide to bind thrombin, suggesting that the two regions must be present in the same polypeptide chain for this effect to occur. These results with isolated HCII 1-75 and 54-75 are consistent with those obtained when these sequences were fused to α_1_-PI, in that fusion protein HAPI M358R (containing HCII 1-75) inhibited thrombin at a rate 3.4-fold more rapid than fusion protein H_55-75_API M358R (containing HCII 55-75) [19].

**Figure 7:**
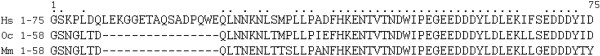
**Alignment of N-terminal primary structures of human, rabbit, and mouse HCII.** The N-terminal 75 residues of human (Homo sapiens, Hs) HCII are shown, aligned with the corresponding residues from the other two mammalian HCII sequences (rabbit, Oryctolagus cuniculus, Oc, and mouse, Mus muscularis, Mm). Dashes indicate no corresponding residue, while dots indicate that the residue is conserved across all three species.

Alignment of human, rabbit, and mouse HCII primary sequences (Figure 7) illustrates that human HCII contains 17 amino acids not found in the N-terminal regions of the other mammalian HCII proteins; residues 8-24 of human HCII have no counterpart in the other two sequences. Assuming that the other HCII orthologues exhibit the same mechanism of action as their human counterpart, and that their far N-terminal sequences would also exert a conformational effect on residues 54-75, then the residues exerting a conformational influence over HCII 54-75 in HCII 1-75 and in fusion protein HAPI M358R should lie between residues 25 and 53 of HCII. Residues 1-8 can be discounted in this analysis given their relatively low degree of identity.

**Figure 8:**
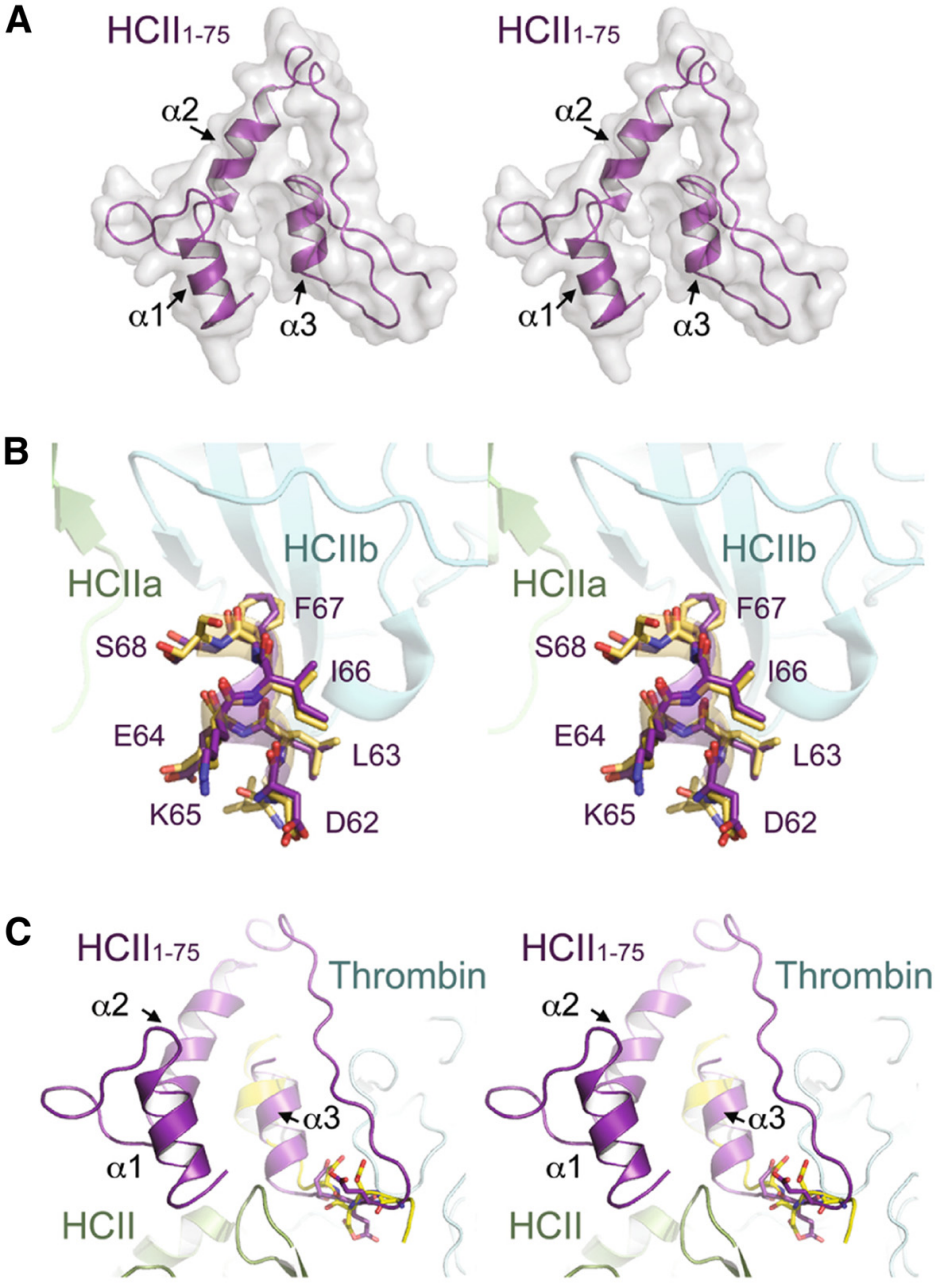
**HCII 1-75 molecular model.** (**A**) Molecular model of HCII residues 1-75. HCII 1-75 is shown in ribbon representation (purple) and corresponding space filling model (grey). Helices are labeled 1, 2 and 3 corresponding to residues 4-12, 26-36 and 60-67 of HCII, respectively. (**B**) Structural superimposition of helix 3 (purple) from the HCII 1-75 molecular model with the same region of HCII (yellow) observed in the apo-HCII crystal structure (PDB 1JMJ). HCII subunits are labeled HCIIa and HCIIb and colored green and cyan, respectively. Amino acid residues of HCII included in the structural alignment are labeled. (**C**) Structural superimposition of HCII 1-75 molecular model with the HCII-thrombin encounter complex determined by x-ray crystallography (PDB 1JMO). HCII, light green; HCII residues 54-73 are shown in yellow; thrombin, cyan; HCII 1-75, purple; acidic residues used for structural alignment are shown in stick. Stereogram images are shown in all cases.

Molecular modeling was employed to test these deductions. As shown in Figure 8A, the modeled HCII 1-75 structure, obtained using a threading algorithm dependent on secondary structure predictions, contains random coiled regions interspersed between three predicted α helices encompassing residues 4-12, 26-36, and 60-67. In neither the HCII dimer crystal structure nor the HCII-thrombin S195A crystallized encounter complex are residues N-terminal to Leu61 visible [20]; however, it was possible to validate the model to a certain extent by demonstrating that it fit the observed structures in the section of the acidic tail C-terminal to residue 61. As shown in Figures 8A and 8B, the model structure demonstrated excellent agreement with both the native HCII and HCII encounter complex crystal structure with respect to helix 3, and, in the latter case, the thrombin: HCII interface. In this context, the model predicts an interaction between helices 2 and 3, which are in a similar plane in three dimensions, as opposed to helix 1, which is canted forward in the stereograms depicted in Figure 8. The predicted interaction of residues 26-36 with residues 60-67 is consistent with our findings both with small polypeptides comprising HCII 1-75 and HCII 54-75 (this study) and with fusion proteins in which these portions of the acidic tail were fused with α_1_-PI M358R [19]. Why this effect has not been noted with truncation mutants of recombinant HCII expressed in either bacterial or baculoviral expression systems [7,44] is not clear. Deletion of residues 1-52 did not change the rate constants for thrombin inhibition either with or without GAG catalysis with untagged recombinant HCII produced using these systems. However, deletion of the first 52 amino acids of recombinant HCII led to a statistically significant, 2-fold reduction in the rate of thrombin inhibition, when wild-type and deletion mutant were both furnished with a C-terminal hexahistidine tag to facilitate purification of the baculovirally expressed proteins [44]. It is also possible that helix 3 is indirectly stabilized by other interactions involving residues in HCII C-terminal to the acidic tail, in the context of truncation mutants of HCII, such that the contribution of the putative helix2 – helix 3 interaction can be best observed when studying the acidic extension in the non-native context.

The relative contributions to HCII function of a template mechanism, in which thrombin and HCII become co-localized due to their binding to the same glycosaminoglycan chain, or a conformational change mechanism, in which GAG binding to HCII releases the N-terminal extension of HCII for exosite 1 binding, remain somewhat controversial [12,15], as does the degree of cross-talk between thrombin’s exosites and active site [45-47]. Nevertheless, reported K_D_ values for thrombin binding to dermatan sulfate (2-6 μM) [47] are much less than that for the HCII-dermatan sulfate interaction (236 – 290 μM) [47] but higher than those reported in this study for HCII 1-75 binding to exosite 1 (0.34 μM). Even if, as was the case with the hirudin dodecapeptide, the lack of tyrosine sulfation on HCII 1-75 caused us to overestimate the 1-75: exosite 1 affinity in native HCII by 5- to 10-fold, this would not change the relative order of affinities. The same order of affinities has been reported when considering high molecular weight heparin rather than dermatan sulphate as the GAG [47]. These relative binding affinities support a mixed mechanism in which thrombin binds GAG and HCII then binds to the thrombin-GAG complex, undergoing a conformational change which liberates HCII 1-75, which then binds thrombin via exosite 1 more tightly than either reactant binds to GAGs, setting the stage for physiologically irreversible inhibition of thrombin via the serpin suicide-substrate mechanism.

## Conclusions

In this study we have characterized the thrombin exosite 1 ligand, the N-terminal acidic extension of HCII, to a degree not previously possible, by expressing it in isolated form. It bound thrombin with a higher affinity than GAGs, as indicated by its K_D_ for thrombin binding of 330-340 nM, and bound more effectively than HCII 54-75; HCII 1-53 did not bind to thrombin. Molecular modelling suggested a structural explanation for a contribution of residues N-terminal to residue 52 in stabilizing the more C-terminal portions of this unique serpin acidic tail; we hypothesize that this model reflects the structure not only of isolated HCII 1-75 but also that region of the intact protein in GAG-activated form. The latter working hypothesis may be tested in future studies by site-directed mutagenesis, now that procedures have been established for expression, purification, and characterization of this exosite-1- binding motif as an isolated polypeptide.

## Abbreviations

α1-PI: α_1_-proteinase inhibitor, α_1_-antitrypsin; α1-PI M358R: α_1_-PI with the substitution of Met358 by Arg; AT: Antithrombin; γT-thrombin: Proteolytic fragment of α-thrombin formed by digestion with trypsin; BSA: Bovine serum albumin; FPR-ck: D-Phe-L-Pro-L-Arg chloromethylketone, active site inhibitor of thrombin; HAPI: Fusion protein of residues 1-75 of heparin cofactor II and all of α_1_-PI; HAPI M358R: HAPI with the M358R substitution; GAG: Glycosaminoglycan; HCII: Heparin cofactor II; HCII 1-75: Recombinant peptide containing the first 75 residues of human HCII and a nonapeptide N-terminal tag; Hir 55-65: N-acetylated synthetic peptide containing the C-terminal 11 residues of hirudin; HCII 1-53: Synthetic peptide containing the first 53 residues of human HCII and a nonapeptide N-terminal tag; HCII 54-75: Synthetic peptide containing the residues 54-75 of human HCII and a nonapeptide N-terminal tag; k2: Second-order rate constant of inhibition; KD: Equilibrium binding constant; Ki: Binding constant of inhibition; K2: RCL, reactive centre loop; serpin: Serine protease inhibitor; P1-P1': The reactive centre peptide bond, where P1 is the amino acid N-terminal to cleavage and P1' is the amino acid C-terminal to cleavage; PBS: Phosphate-buffered saline; PBS-T: PBS containing 0.1% Tween-20; SDS: Sodium dodecyl sulfate; SDS-PAGE: SDS polyacrylamide gel electrophoresis; sulfo-Hir 54-65: Synthetic peptide containing the C-terminal 12 residues of hirudin, with Tyr63 sulfated; WT: Wild-type.

## Competing interests

The authors declare that they have no competing interests.

## Authors’ contributions

WPS conceived of the study, secured competitive funding, directed experiments, and wrote the manuscript. AJB, LAR, VB, and MDL performed all *in vitro* and *in vivo* experiments and developed and refined experimental protocols. MSJ provided structural biology expertise and advice and performed the molecular modeling work. PCL and JIW provided input into the design and revision of the experimental plan. All authors participated in editing and revising the manuscript. All authors read and approved the final manuscript.
